# Sex differences in congenital hereditary endothelial dystrophy (CHED) and *Slc4a11*^−/−^ mouse model of CHED

**DOI:** 10.1186/s13293-026-00879-9

**Published:** 2026-03-28

**Authors:** Wenlin Zhang, Divya Sree Ramya, Eduardo Araujo, Jhuwala Venkatakrishnan, Saloni Gupta, Isa Matsubayashi, Marco Morselli, Ananya Kaginalkar, Sunita Chaurasia, Matteo Pellegrini, Radhika Tandon, Muralidhar Ramappa, Arthur Arnold, Anthony J. Aldave

**Affiliations:** 1 Stein Eye Institute, David Geffen School of Medicine at UCLA, UCLA, University of California Los Angeles, 200 Stein Plaza, Los Angeles, CA 90095-7003 USA; 2 Jasti V Ramanamma Children’s Eye Care Center, The Cornea Institute, & Institute for Rare Eye Diseases and Ocular Genetics L.V. Prasad Eye Institute, Hyderabad, Telangana India; 3 Department of Human Genetics, University of California Los Angeles, Los Angeles, CA 90095 USA; 4 Department of Molecular, Cell & Developmental Biology, University of California Los Angeles, Los Angeles, CA USA; 5 Dr. Rajendra Prasad Centre for Ophthalmic Sciences, All India Institute of Medical Sciences, New Delhi, India; 6 Department of Integrative Biology & Physiology, University of California Los Angeles, Los Angeles, CA USA

**Keywords:** CHED (Congenital Hereditary Endothelial Dystrophy), Solute carrier family 4 member 11 gene (*SLC4A11*), Sex differences, Corneal edema, *Slc4a11*^-/-^ mouse model, Estrogen, Oxidative stress, Lipid β-oxidation, Transcriptomic analysis, Gene therapy

## Abstract

**Background:**

Sex differences have been described in several corneal diseases such as Fuchs endothelial corneal dystrophy and keratoconus, with estrogens implicated in the induction of these differences. Here, we report the identification of sex differences in a cohort of 177 individuals with Corneal Hereditary Endothelial Dystrophy (CHED), a rare corneal endothelial dystrophy associated with biallelic *SLC4A11* gene mutations, and in a *Slc4a11*^−/−^ mouse model of CHED.

**Methods:**

Central corneal thickness (CCT) was measured in individuals with CHED and in *Slc4a11*^−/−^ and *Slc4a11*^+/+^ mice to identify a correlation between sex and the degree of corneal edema. To investigate potential causes of such a correlation, RNAseq analysis and mitochondrial superoxide measurement were performed on corneal endothelium from male and female *Slc4a11*^−/−^ and *Slc4a11*^+/+^ mice, for which body composition analysis was also performed. Gonadectomy or sham surgery was performed in *Slc4a11*^−/−^ and *Slc4a11*^+/+^ mice at 4 weeks of age with subsequent longitudinal CCT and body weight monitoring, followed by an analysis of the interaction effect of surgery type, sex and genotype on CCT.

**Results:**

Male sex is associated with increased CCT, and thus more severe corneal edema, the characteristic clinical feature of CHED, in affected individuals and *Slc4a11*^−/−^ mice. The corneal endothelium in male *Slc4a11*^−/−^ mice demonstrates increased levels of oxidative stress compared to *Slc4a11*^−/−^ female mice, as evidenced by higher levels of glucose- and glutamine-derived mitochondrial superoxide, controlling for age. Removal of gonadal hormones in *Slc4a11*^−/−^ mice increases corneal edema in female mice, suggesting a protective role for ovarian hormones. Transcriptomic analysis of corneal endothelium and body composition analysis in *Slc4a11*^+/+^ and *Slc4a11*^−/−^ mice suggest that estrogens play a role in promoting corneal endothelial utilization of lipids via β-oxidation as an alternative energy source in the absence of SLC4A11-mediated NH_3_:H transport function, thereby reducing oxidative stress from glucose and glutamine metabolism.

**Conclusions:**

Male sex is associated with a more severe corneal phenotype in individuals with CHED and a *Slc4a11*^*−/−*^ mouse model of the disease. Increased corneal edema in female *Slc4a11*^*−/−*^ mice following gonadectomy suggests ovarian hormones play a protective role in maintaining corneal deturgescence in the setting of loss of SLC4A11 function.

**Supplementary Information:**

The online version contains supplementary material available at 10.1186/s13293-026-00879-9.

## Abstract (plain language summary)

Corneal Hereditary Endothelial Dystrophy (CHED) is a rare inherited eye disease that is associated with blurred vision that develops in newborns, infants and children due to swelling and clouding of the normally clear corneas. To be able to study the disease and identify potential treatments for it, a mouse model of the disease has been created that closely resembles the disease seen in affected individuals. We identified a sex difference in disease severity in both affected individuals and mice, with males demonstrating more severe corneal swelling than females. A possible explanation for this difference was the discovery that the corneas of affected male mice had higher levels of oxidative stress, which can lead to damage of the cells of the cornea. To determine the potential role of hormones in the observed sex differences between the male and female individuals and mice, the organs that produce hormones (gonads) were removed at a young age in the affected mice. Removal of the ovaries in the female mice, leading to a loss of the female hormones, led to a worsening of the corneal swelling. This suggests that the female hormones, which include estrogen, prevent swelling of the corneas in affected mice, and possibly in affected female newborns, infants and children.

**Supplementary Information:**

The online version contains supplementary material available at 10.1186/s13293-026-00879-9.

## Highlights

- This study is the first to identify sex-based differences in the severity of corneal edema in individuals with CHED, made possible by analyzing the largest patient cohort ever reported, and in a *Slc4a11*^-/-^ mouse model of CHED.
- Male newborns, infants and children with CHED and male *Slc4a11*^-/-^ mice have significantly worse corneal edema than affected female newborns, infants and children and *Slc4a11*^-/-^ mice.
- Sex differences between male and female mice were identified at the phenotypic (corneal edema), functional (oxidative stress) and transcriptomic (corneal endothelial gene expression) levels.
- Removal of gonadal sex hormones by gonadectomy in female *Slc4a11*^-/-^ mice worsens corneal edema, suggesting ovarian hormones protect against progressive loss of corneal endothelial cell function in the setting of loss of SLC4A11.

**Supplementary Information:**

The online version contains supplementary material available at 10.1186/s13293-026-00879-9.

## Background

**Fig. 1:**
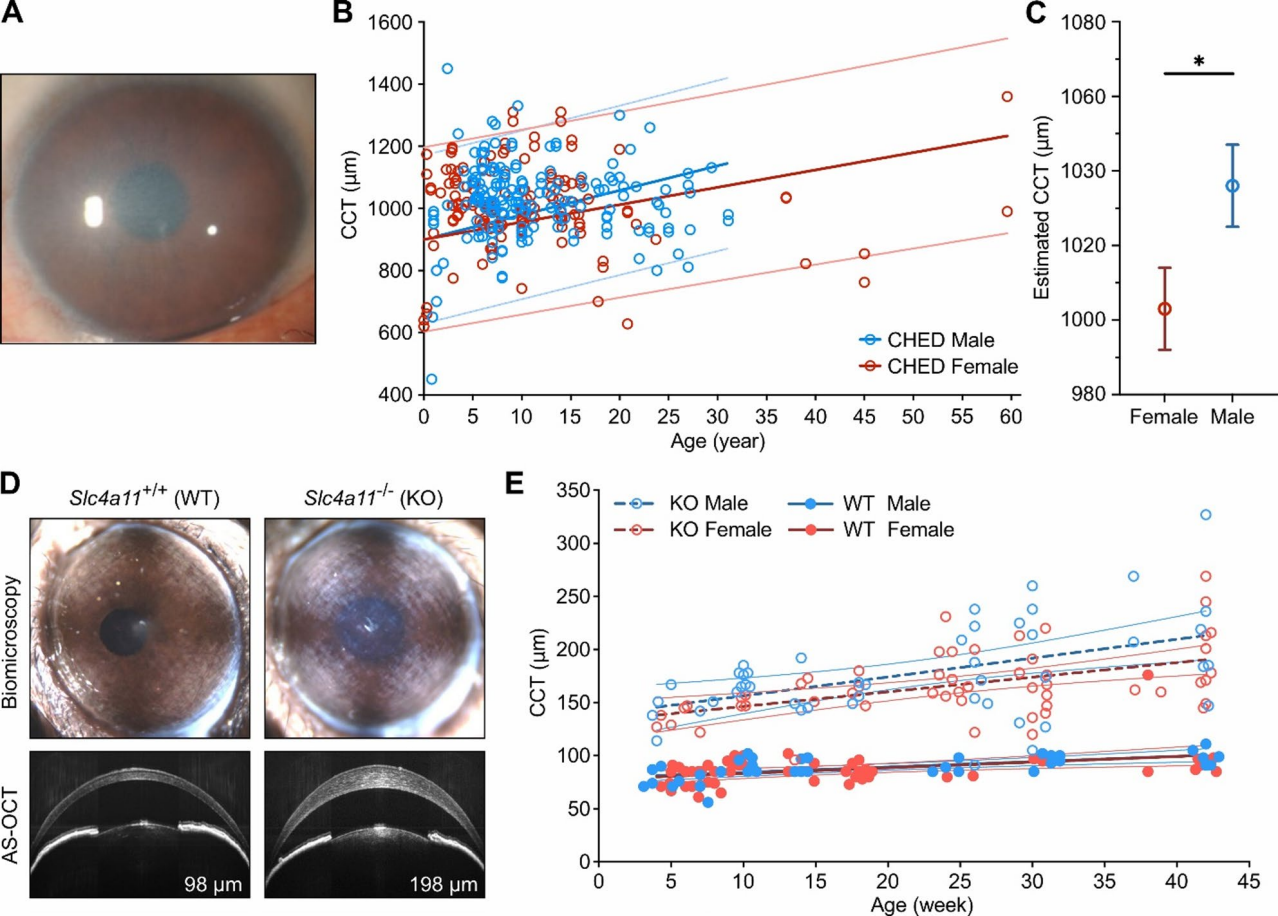
Sex differences of corneal edema in CHED and *Slc4a11*^*−/−*^ mice. (**A**) Slit lamp photo showing diffuse corneal edema in a 30-year-old woman with CHED harboring biallelic *SLC4A11* mutations. (**B**) Central corneal thickness (CCT) in individuals with CHED plotted against age and separated by sex. Fitted linear curves (solid lines) with 95% CI (dotted lines) of predicted CCT are also plotted. (**C**) Estimated CCT in male and female individuals with CHED at 11 years of age. Estimated mean and SEM are plotted. (**D**) Representative in vivo slit lamp biomicroscopy and anterior segment ocular coherence tomography (AS-OCT) images of 24-week-old *Slc4a11*^+/+^ (WT) and *Slc4a11*^−/−^ (KO) mice, showing diffuse corneal edema and increased CCT in KO mice. (**E**) CCT in KO and WT mice plotted against age and separated by sex. Fitted linear curves (dashed bold lines - KO, solid bold lines - WT) with 95% CI (fine lines) are also plotted. * *p* < 0.05

Congenital Hereditary Endothelial Dystrophy (CHED, OMIM# 217700, ORPHAcode 293603) is an autosomal recessive disorder of the corneal endothelium, characterized by bilateral corneal edema and loss of corneal clarity presenting at birth or in the first decade of life (Fig. 1A). The diffuse corneal stromal edema is progressive and may be associated with increased corneal thickness up to twice normal. Symptoms consist of bilateral visual impairment, which is typically significant, and secondary deprivational amblyopia and nystagmus. CHED is one of the most frequently encountered corneal dystrophies in regions in which consanguineous marriages are more common (Middle East, South Asia, and Southeast Asia), and is a well-recognized cause of congenital corneal edema, estimated to affect three in 100,000 newborns in Europe [1]. The selection of CHED as one of eight rare genetic disorders in The Accelerating Medicines Partnership^®^ (AMP^®^) Program Bespoke Gene Therapy Consortium (AMP BGTC), with the goal of developing adeno-associated virus (AAV) gene therapy for CHED, necessitates a better understanding of the natural history of the disorder, and the effect of relevant biologic variables such as sex on the disease phenotype and response to AAV gene therapy [2].

CHED is associated with biallelic coding region mutations in the solute carrier family 4 member 11 gene (*SLC4A11*) in approximately 80% of affected individuals screened to date [3, 4]. In the C57BL/6 *Slc4a11*^−/−^ mouse model of CHED, which contains a targeted deletion of exons 9–13 of the murine *Slc4a11* gene, characteristic corneal findings reminiscent of those observed in individuals with CHED are observed, including progressive corneal edema and thickening of Descemet membrane [5]. SLC4A11 is one of the most highly expressed differentiation markers for corneal endothelial cells [6–8], and is essential in energy-producing glutaminolysis by maintaining ammonia homeostasis, reducing glutaminolysis-associated oxidative stress, maintaining antioxidant signaling, and preventing apoptosis in the corneal endothelium [9–13]. In addition, multiple lines of evidence support dysfunction of the corneal endothelial “pump” activity secondary to loss of SLC4A11 function as the direct cause of corneal edema in CHED and *Slc4a11*^−/−^ mice [14–20]. Long-term changes in the corneal endothelial cells resulting from SLC4A11 loss of function include generalized inhibition of cellular metabolic activities, accumulation of mitochondrial reactive oxygen species [21], aberrations in actin cytoskeleton assembly and tight junction organization, increased paracellular permeability, and apoptosis-mediated cell death [10–13, 15, 16, 22].

Sex differences have been described in several of the most common inherited corneal disorders such as Fuchs endothelial corneal dystrophy (FECD) and keratoconus. Age-related FECD has a greater incidence in women than in men, with a reported ratio of 3:1 to 4:1 [23, 24], and the female sex is the most significant risk factor for developing advanced FECD in addition to age [25]. The effect of estrogens has been postulated to account for the increased incidence and more severe phenotype of FECD in women [25]. Studies in an ultraviolet A (UVA) light-induced murine model of FECD suggest the upregulation of estrogen-metabolizing enzyme CYP1B1 and the formation of reactive estrogen metabolites and estrogen-DNA adducts in female mice are the driving force for the sex-dependent differential response to UVA in the corneal endothelium [26], supported by the demonstration that inhibition of CYP1B1 rescues corneal endothelial loss [27]. Another study in cultured primary human corneal endothelial cells from healthy donors showed exposure to a supraphysiologic concentration of 17-β estradiol (E2) was associated with mitochondrial fragmentation and decreased cellular ATP levels only in cells from female donors [28]. 

Interestingly, heterozygous *SLC4A11* mutations have also been associated with FECD [4], with 28 distinct mutations reported and accounting for about 11% of individuals with FECD [4, 29–34]. In contrast, keratoconus is associated with a higher prevalence, a younger age at initial presentation, and a higher percentage of eyes with an advanced phenotype in men [35, 36]. A study performed in cultured human corneal stromal cells suggests that E2-mediated downregulation of collagen-degrading matrix metalloproteinase-2 and upregulation of alpha1-proteinase inhibitor may serve protective roles against keratoconus development in women [37].

In this study, we evaluated sex as a biological variable in the clinical phenotype of newborns, infants and children with CHED and *Slc4a11*^−/−^ mice. We then explored the possible cause of such sex-related differences via transcriptomic analysis and functional characterization. Subsequently, we investigated the role of gonadal hormones in inducing the apparent sex difference by performing gonadectomy in *Slc4a11*^+/+^ and *Slc4a11*^−/−^ mice and a potential mechanism for the protective role of gonadal estrogens in preserving corneal clarity in *Slc4a11*^−/−^ mice was identified based on transcriptomic and functional analyses.

## Methods

### Sex as a biological variable

This study investigated the sex differences in newborns, infants and children affected with CHED and in a *Slc4a11*^−/−^ mouse model of CHED. Therefore, the study was designed to account for sex as a biological variable by including a balanced number of both sexes in each of the analyses involving human subjects and in each of the studies performed using *Slc4a11*^−/−^ and *Slc4a11*^+/+^ mice.

### Corneal thickness measurement in infants and children with CHED

Clinical information regarding the age of diagnosis, sex, central corneal thickness (CCT, as a measure of corneal edema) measured by anterior segment optical coherence tomography (AS-OCT), and the age at the time of CCT measurement was obtained for 177 individuals with CHED, including 73 published cases (39 male, 17 female) and 104 unpublished cases (57 male, 47 female) [14, 38–58]. CCT measurements performed in unoperated eyes, along with the age at which the CCT measurements were obtained, were recorded and analyzed.

### *Slc4a11*^−/−^ mouse model

*Slc4a11* ^−/−^ C57BL/6 mice with a targeted deletion of exons 9 to 13 of the murine *Slc4a11* gene were used in this study [59]. Mice were maintained on a standard chow diet and on a 12 h light/dark cycle from 6 am to 6 pm at ambient temperature (~ 22 °C) with controlled humidity (~ 45%) in pathogen-free conditions. Heterozygous (*Slc4a11*^*+/−*^) mice were intercrossed to generate littermates of homozygous *Slc4a11*^+/+^ (WT) and *Slc4a11*^−/−^ (KO) mice. All mice were genotyped at weaning from tail biopsies using real time PCR by a commercial vendor (Transnetyx).

### Corneal thickness measurement in *Slc4a11*^−/−^ mice

Mice were anesthetized with an intraperitoneal injection of ketamine/xylazine 0.1/0.01 mg/mg per gram body weight and imaged using the Bioptigen OCT system with an anterior segment lens (Leica Microsystems). OCT images were manually analyzed to obtain CCT measurements.

### Body weight, body composition, and tissue collection in *Slc4a11*^−/−^ and *Slc4a11*^+/+^ mice

Mice were typically fasted for 4 h before body weight measurement, body composition analysis, and tissue collection. Body composition was determined by a Mouse Minispec apparatus (Bruker Woodlands, TX) with Echo Medical Systems (Houston, TX) software, which uses nuclear magnetic resonance (NMR) spectroscopy for fat and lean mass measurements, as described previously [60]. Parameters measured include fat mass (g), lean muscle mass (g), free H_2_O (g) and total H_2_O (g). Following euthanization, liver, bilateral kidney, spleen, bilateral subcutaneous inguinal white adipose tissue (iWAT), one side of gonadal white adipose tissue (gWAT), brown adipose tissue (BAT), and tibia bone were collected, weighed and measured.

### RNAseq of ex vivo corneal endothelium from *Slc4a11*^−/−^ and *Slc4a11*^+/+^ mice

Following euthanization of *Slc4a11*^-/-^ and *Slc4a11*^+/+^ mice at 10 weeks of age (3 males and 3 females each), each globe was removed, and the Descemet membrane was separated from the posterior surface of each cornea. The Descemet membranes from both corneas of *Slc4a11*^-/-^ mice and *Slc4a11*^+/+^ mice were immediately processed as pairs for total RNA isolation with genomic DNA digestion using RNeasy Plus Micro Kit (Qiagen). Purified total RNA was then prepared for RNAseq libraries using SMARTer^®^ Stranded Total RNA-Seq Kit v2 - Pico Input Mammalian (634411, Takara Bio USA). Libraries were sequenced on Novaseq S1 and paired-end 50-bp reads were generated.

### RNAseq data analysis

Raw reads were aligned to mouse genome GRCm38/mm10 using Hisat2. Raw counts of aligned reads were converted to counts per million (CPM) mapped reads and normalized by the method of trimmed mean of M values (TMM) to adjust for library size differences. Linear models for microarray analysis (LIMMA) coupled with variance modeling at the observation level (VOOM) were used for differential gene expression analysis. The CPM fold change of gene transcripts was calculated in a 2-genotype by 2-sex factorial design matrix, testing the effect of genotype, the effect of sex, and the interaction of the two. The following thresholds were applied to define genes with differential expression: CPM > 1, log_2_ fold change |log_2_FC| > 1, and adjusted p-value < 0.05. A differential gene expression (DGE) list was created for each testing term, including genotype (WT vs. KO), sex (male vs. female), and genotype*sex interaction (sexually dimorphic differential gene expression).

### Ingenuity pathway analysis

Ingenuity Pathway Analysis (IPA^®^, QIAGEN Bioinformatics) was used to predict upstream regulators given observed differential expression. Upstream regulators with a predicted activation z-score > 2 or <−2 were retained. A positive “activation z-score” predicts an activation of the regulator-mediated pathway, whereas a negative “activation z-score” predicts an inhibition of the pathway. The lists of differentially expressed genes in each identified regulator’s pathway were subsequently obtained.

### Mitochondria superoxide measurement in corneal endothelium of *Slc4a11*^−/−^ mice

Mitochondrial superoxide (Mito-O_2_^•−^) was measured using liquid chromatography-mass spectrometry (LC-MS, Agilent 6545 Q-TOF) based on published protocols with modifications for corneal endothelial samples [61–63]. In brief, Descemet membrane was peeled from freshly dissected corneas of *Slc4a11*^+/+^ and *Slc4a11*^−/−^ mice at the indicated ages, with bilateral Descemet membrane samples from the same mouse placed together as a pair. Descemet membrane pairs were incubated in Krebs-Henseleit bicarbonate buffer (5.5 mM glucose) or glutamine bicarbonate buffer (5.5 mM glutamine) at 37 °C in dark with 3 µM MitoSOX™ Red (or Mito-HE, M36008, Invitrogen) for 30 min, and then washed in ice-cold PBS solution two times before being snap frozen in PBS with 1% (v/v) Triton X-100 in liquid nitrogen. The detailed composition of Krebs-Henseleit bicarbonate buffer and glutamine bicarbonate buffer are in Supplemental Table 1. The frozen Descemet membrane pairs were then homogenized with a tissue grinder on ice while protected from light and centrifuged at 600 *g* at 4 °C. The supernatants were mixed with − 20 °C acetone at a 1:4 volume ratio to allow protein precipitation for 60 min at −20 °C. The protein precipitate was then removed by centrifugation at 20,000 *g* for 10 min at 4 °C. The supernatant was assessed for O_2_^•−^ specific MitoSOX oxidation product 2-dihydroethidiumm (2-OH-Mito-E^+^) by LC-MS. The metabolite identification was based on correct retention time and matching multiple reaction monitoring (MRM) ion ratios of pure metabolites as previously described [61]: Mito-E^2+^ (m/z 315.7, retention time 6.97 min) and 2-OH-Mito-E^2+^ (m/z 323.7, retention time 6.97 min). Peak integration was performed using Agilent MassHunter software (Agilent). All peaks were reviewed manually and adjusted if necessary. The peak area of the first transition per metabolite was used for subsequent calculations of the level of 2-OH-Mito-E^2+^ normalized to Mito-E^2+^ in each Descemet membrane pair to yield 2-OH-Mito-E^2+^%. Example LC-MS chromatograms showing the m/z transitions of samples of MitoSOX probe only, Descemet membrane only, and Descemet membrane with MitoSOX are in Supplemental Fig. 1.

### Gonadectomy in *Slc4a11*^−/−^ and *Slc4a11*^+/+^ mice

At 4 weeks of age, *Slc4a11*^−/−^ and *Slc4a11*^+/+^ mice underwent gonadectomy, with sham-operated mice serving as controls. The gonadectomy was performed under isoflurane anesthesia (1–2% isoflurane in 95% oxygen) and followed standard aseptic surgery procedure. For male mice, the scrotal skin was incised at midline, the bilateral testes were removed, and the incisions were closed with wound clips. For female mice, the ovaries were removed through bilateral incisions in the skin and abdominal muscle wall at locations just below the bilateral rib cages (about midway between the hip and the bottom of the rib cage, and about 2/3 of the way from ventrum to dorsum). The abdominal muscle layer was then sutured, and the skin incisions were closed with wound clips. In sham-operated control mice, incisions were made, the gonads were briefly manipulated but remained intact, and the incision was closed as described above. After surgery, CCT was measured monthly, and body weight was measured biweekly for 5 months. All gonadectomized mice underwent autopsy after the last data collection point following euthanization to confirm the absence of bilateral gonads.

### Primary human corneal endothelial cell culture and knock-down of SLC4A11

Primary cultures of human corneal endothelial cells (pHCEC) were established from donor corneas as previously described [64]. After achieving a confluent endothelial cell monolayer, passage 1 (p1) of pHCEC were transfected with 10 nM anti-SLC4A11 siRNA (CCGAAAGUACCUGAAGUUAAAGAACT) or scrambled siRNA (OriGene Technologies) using Lipofectamine LTX (Life Technologies). At 72 h post-transfection, the cells were lysed for protein isolation.

### Capillary electrophoresis-based Western Blot

Whole-cell lysates from pHCEC were prepared with RIPA buffer with proteinase and phosphatase inhibitors. Total protein was quantified by the bicinchoninic acid (BCA) assay, separated, and detected on Simple Western system Wes^™^ (ProteinSimple). Quantification and data analysis were performed in Compass for SW software (ProteinSimple). Antibodies used include anti-SLC4A11 antibody (custom made), anti-AMPKα antibody (Cell Signaling, 5831), anti-phospho-AMPKα (Thr173) antibody (Cell Signaling, 2535), anti-Acetyl-CoA Carboxylase (ACC) antibody (Cell Signaling, 3676), and anti-phospho-ACC (Ser79 in ACC1, Ser221 in ACC2) antibody (Cell Signaling, 3661).

### Statistical analysis

All descriptive data were plotted as dot plots showing individual data points, with the mean ± SEM depicted as a line with an error bar. Age-dependent CCT change in infants and children with CHED separated by sex, age-dependent CCT change in *Slc4a11*^−/−^ and *Slc4a11*^+/+^ mice separated by sex, and age-dependent CCT and body weight change following GDX in *Slc4a11*^−/−^ and *Slc4a11*^+/+^ mice separated by sex and genotype were analyzed using generalized linear mixed model (GLMM) in SPSS software (IBM), in which CCT or body weight values from the same individual or mouse over time were treated as repeated measures. GLMM-estimated mean CCT and body weight for each sex and genotype group were plotted over a range of ages as a line with standard error bars. For 2-OH-Mito-E^2+^% comparison between different sexes and genotypes, 2-way ANOVA with post-hoc multiple comparisons were used. For parameters measured in body composition analysis, student t-tests were performed comparing male *Slc4a11*^−/−^ to male *Slc4a11*^+/+^ mice and female *Slc4a11*^−/−^ to female *Slc4a11*^+/+^ mice. Statistical analysis of RNAseq data was performed in R using software packages listed under the section “RNAseq data analysis”. In all figures, the following symbols were used to annotate the p values: ns, *p* > 0.05; *, *p* < 0.05; **, *p* < 0.01; ***, *p* < 0.001; ****, *p* < 0.0001.

## Results

### Male sex is associated with more severe corneal edema in newborns, infants and children with CHED and in *Slc4a11*^−/−^ mice

We reviewed the English language published literature for all individuals diagnosed with CHED and collected clinical information regarding the age of diagnosis, sex, preoperative corrected visual acuity and CCT, as a measure of corneal edema, and the age at the time of CCT measurement. Of the 431 individuals (217 male, 214 female) reported to have CHED, 73 individuals (44 male, 29 female) had at least one CCT measurement recorded. In addition, we collected clinical information on 104 previously unpublished individuals with CHED (57 male, 47 female) for whom at least one CCT measurement had been recorded. In this combined cohort of 177 individuals with CHED, there was an equal distribution of male (99, 56%) and female (78, 44%) individuals (Chi-square test, χ^2^ = 2.49, df = 1, *p* = 0.11). The median age at the time of diagnosis was not significantly different in male (7.9 years) versus female (7.5 years) individuals (Independent sample median test, *p* = 0.841).

A total of 340 eyes were included in the final analysis (Fig. 1B), testing for the effect of age and sex on CCT in CHED, using the generalized linear mixed model. The analysis revealed a significant effect of sex (F = 2140.6, df1 = 2, df2 = 336, *p* < 0.001) but a non-significant effect of age (F = 1.5, df1 = 1, df2 = 336, *p* = 0.223) on CCT. Model estimated CCT at age 11 (mean age of all eyes with preoperative CCT) for male and female individuals with CHED were 1036 ± 11 μm and 1003 ± 11 μm, respectively (Fig. 1C, pairwise contrast, F = 4.66, df1 = 1, df2 = 336, *p* = 0.032).

*Slc4a11*^−/−^ (KO) mice demonstrated a progressive increase in corneal edema associated with increasing CCT on AS-OCT from 4 weeks (129 ± 6 μm) to 42 weeks of age (203 ± 12 μm) (Fig. 1D), while *Slc4a11*^+/+^ (WT) mice demonstrated clear corneas and smaller increase in CCT between 4 weeks (78 ± 3 μm) and 42 weeks (96 ± 2 μm). An analysis of the effect of genotype, age, and sex on CCT in 188 eyes (Fig. 1E; WT *n* = 88, KO *n* = 100; female *n* = 97, male *n* = 91) using the generalized linear model revealed genotype (KO vs. WT, F = 493.6, df = 1, *p* < 0.0001) and age (F = 43.3, df = 1, *p* < 0.0001) had significant, independent effects on CCT. While sex also had a significant effect on CCT in KO mice, with more severe corneal edema in male mice compared to age-matched female mice, it did not in WT mice (sex, F = 2.9, df = 1, *p* = 0.089; sex*genotype interaction, F = 4.0, df = 1, *p* = 0.046). The model estimated CCT at 21 weeks (mean age of all eyes included) was 89 ± 4 μm in both WT male and female mice, as compared to 175 ± 4 μm in KO male mice and 161 ± 4 μm in KO female mice.

### Sex differences are evident at transcriptomic and functional levels in corneal endothelium of *Slc4a11*^−/−^ mice

**Fig. 2:**
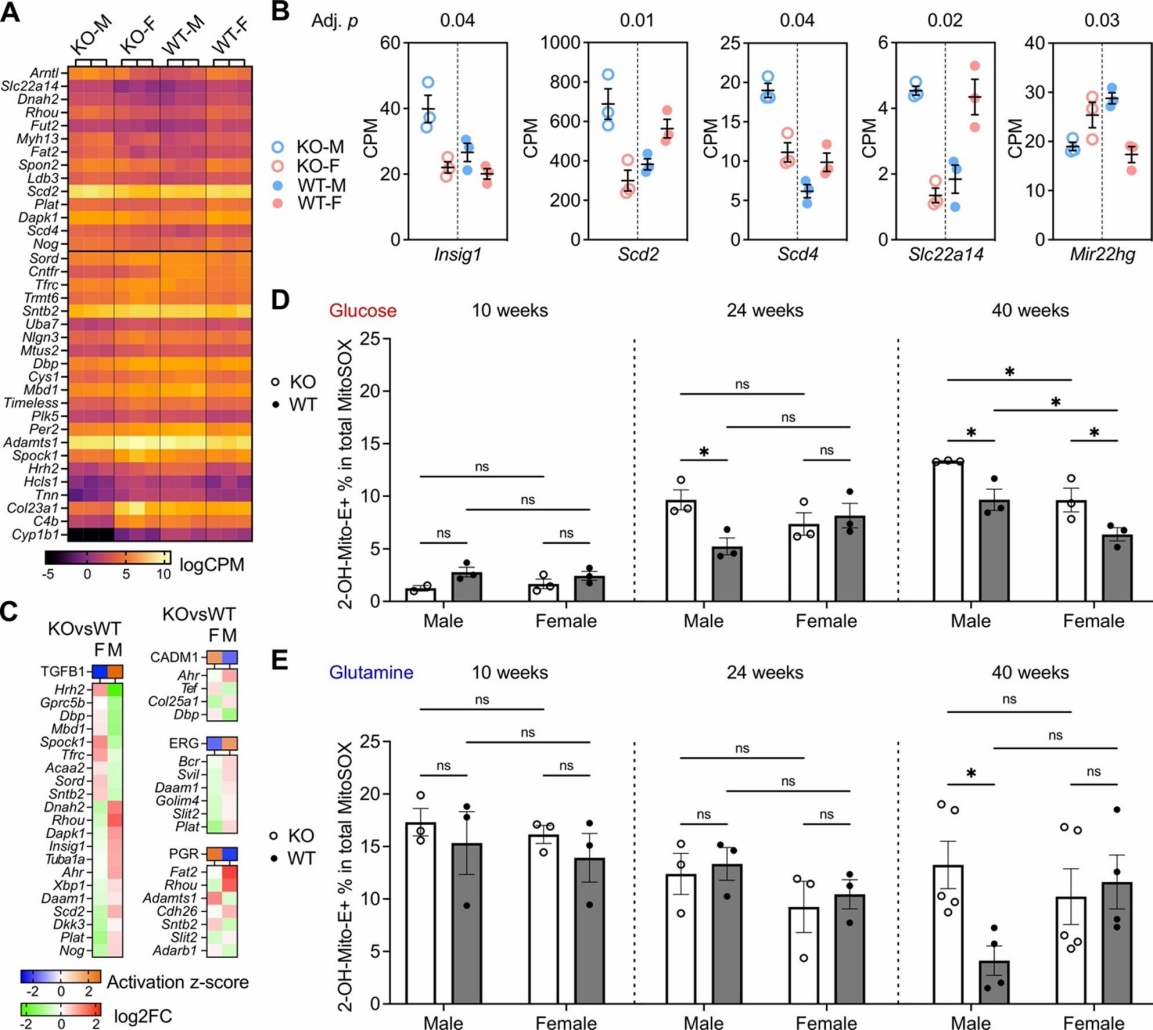
Sex differences in transcriptome and mitochondrial superoxide levels in corneal endothelium of *Slc4a11*^*−/−*^ mice. (**A**) Heatmap showing top sexually dimorphic differentially expressed genes with |log_2_FC| > 1.3, adjusted *p* < 0.05. (**B**) Dot plot of transcript levels (CPM) of *Insig1*, *Scd2*, *Scd4*, *Slc22a14*, and *Mir22hg* genes in CEC of KO male (KO-M) and female (KO-F) mice compared to corneal endothelial cells (CEC) of WT male (WT-M) and female (WT-F) mice. Adjusted *p* values are shown on the top of each plot. (**C**) Heatmaps of IPA-predicted activation z-score for upstream regulators with opposite predicted pathway activity (orange: activation; blue: inhibition) in female (F) and male (M) KO CEC samples compared to WT CEC of the same sex. Below these heatmaps are heatmaps of the log_2_FC of differentially expressed genes in the pathways of the upstream regulators (red: up-regulation; green: down-regulation). (**D**) Bar-dot plots of glucose-dependent Mito-O_2_^•−^ measured by 2-OH-Mito-E^2+^% in KO and WT mice separated by sex at 10, 24 and 40 weeks of age. (**E**) Bar-dot plots of glutamine-dependent Mito-O_2_^•−^ measured by 2-OH-Mito-E^2+^% in KO and WT mice separated by sex at 10, 24, and 40 weeks of age. ns, *p* > 0.05; *, *p* < 0.05

To provide insights into the molecular pathways causing the apparent phenotypic sex differences in individuals with CHED and in *Slc4a11*^−/−^ mice, RNAseq was performed on corneal endothelial cells (CEC) dissected from KO male (*n* = 3), KO female (*n* = 3), WT male (*n* = 3) and WT female (*n* = 3) mice at 10 weeks of age. RNAseq analysis performed in a two-genotype by two-sex factorial design matrix identified genes with a sexually dimorphic differential expression pattern in CEC of KO mice (86 genes with a threshold of |log_2_FC| > 1 and adjusted *p* < 0.05 and 36 genes with a threshold of |log_2_FC| > 1.3 and adjusted *p* < 0.05) by testing genotype*sex interaction (Fig. 2A). Several of the differentially expressed genes encode factors regulating lipid metabolism and have established roles in inducing sex differences: *Insig1*,* Scd2*,* Scd4*,* Slc22a14*, and *Mir22hg* (Fig. 2B). *Insig1* encodes insulin-induced gene 1, a negative regulator of the sterol regulatory element-binding transcription factor 1 (SREBF1) pathway responsible for endogenous cholesterol, fatty acid, triacylglycerol, and phospholipid synthesis, and is regulated by androgen [65, 66]. *Scd2* and *Scd4* encode stearoyl-CoA desaturase 2 and 4, the rate limiting enzymes in *de novo* synthesis of monounsaturated fatty acid, and their expression is regulated by estrogen [67]. *Slc22a14* encodes a mitochondrial riboflavin transporter essential for fatty acid $\beta$-oxidation [68]. *Mir22hg* encodes a long non-coding RNA that gives rise to miR-22, a microRNA that regulates lipid metabolism and is regulated by estrogen in a sexual dimorphic manner [69]. In addition, we observed a borderline significant, sexually dimorphic differential expression of *Srebf1* (log_2_FC = −1.13, adjusted *p* = 0.055), which encodes SREBF1.

Ingenuity Pathway Analysis (IPA) predicted that the top four upstream regulators responsible for the sexually dimorphic differential expression pattern in CEC of KO mice were transforming growth factor-β1 (TGFB1), cell adhesion molecule 1 (CADM1), erythroblast transformation specific-related transcription factor (ERG) and progesterone receptor (PGR). Specifically, there was predicted inhibition (negative “activation z-score”) of TGFB1- and ERG-mediated pathways in female KO CEC but predicted activation (positive “activation z-score”) of TGFB1- and ERG-mediated pathways in male KO CEC. In contrast, there was predicted activation of CADM1- and PGR-mediated pathways in female KO CEC but predicted inhibition of CADM1- and PGR-mediated pathways in male KO CEC (Fig. 2C).

As loss of SLC4A11 function in CEC was previously associated with accumulation of mitochondria reactive oxygen species [13], we sought to determine if there were any sex differences in the mitochondria superoxide (Mito-O_2_^•−^) levels in CEC of *Slc4a11*^−/−^ mice. Mito-O_2_^•−^ levels were quantified in CEC dissected from KO and WT mice at ages 10-, 24-, and 40-weeks following incubation with MitoSOX under glucose-only (5.5 mM) or glutamine-only (5.5 mM) conditions by measuring the percentage of oxidized MitoSOX (2-OH-Mito-E^2+^ %) to total MitoSOX. In the setting of glucose-dependent energy production (Fig. 2D), CEC from male KO mice showed significantly increased 2-OH-Mito-E^2+^% compared to CEC from male WT mice at 24 weeks (2-way ANOVA, Sex*Genotype interaction, F = 20.3, df = 1, *p* = 0.032; post-hoc Sidak’s multiple comparison Male KO vs. Male WT adjusted *p* = 0.014) and at 40 weeks (2-way ANOVA, Genotype F = 35.6, df = 1, *p* = 0.003, Sex F = 36.5, df = 1, *p* = 0.0028; post-hoc Sidak’s multiple comparison Male KO vs. Male WT adjusted *p* = 0.027). In comparison, CEC from female KO mice showed significantly increased 2-OH-Mito-E^2+^% compared to CEC from female WT mice at 40 weeks (post-hoc Sidak’s multiple comparison Female KO vs. Female WT adjusted *p* = 0.047) but not at 24 weeks (post-hoc Sidak’s multiple comparison Female KO vs. Female WT adjusted *p* = 0.83). When comparing CEC from male and female KO mice, significantly higher levels of 2-OH-Mito-E^2+^% were present in CEC from male KO mice at 40 weeks (post-hoc Sidak’s multiple comparison Male KO vs. Female KO, adjusted *p* = 0.0078).

In the setting of glutamine-dependent energy production (Fig. 2E), CEC from male KO mice demonstrated significantly increased 2-OH-Mito-E^2+^% compared to CEC from male WT mice at 40 weeks (2-way ANOVA, Sex*Genotype interaction, F = 122.7, df = 1, *p* = 0.0427; post-hoc Sidak’s multiple comparison Male KO vs. Male WT adjusted *p* = 0.032), while CEC from female KO mice did not demonstrate increased 2-OH-Mito-E^2+^% at 10, 24 or 40 weeks when compared to CEC from female WT mice (40 weeks, post-hoc Sidak’s multiple comparison Female KO vs. Female WT adjusted *p* = 0.90). In summary, loss of SLC4A11 function in CEC of male KO mice resulted in increased levels of mitochondrial superoxide when compared to CEC of male WT and female KO mice.

### Ovarian hormones serve a protective role in preserving corneal thickness and clarity in *Slc4a11*^−/−^ mice

**Fig. 3:**
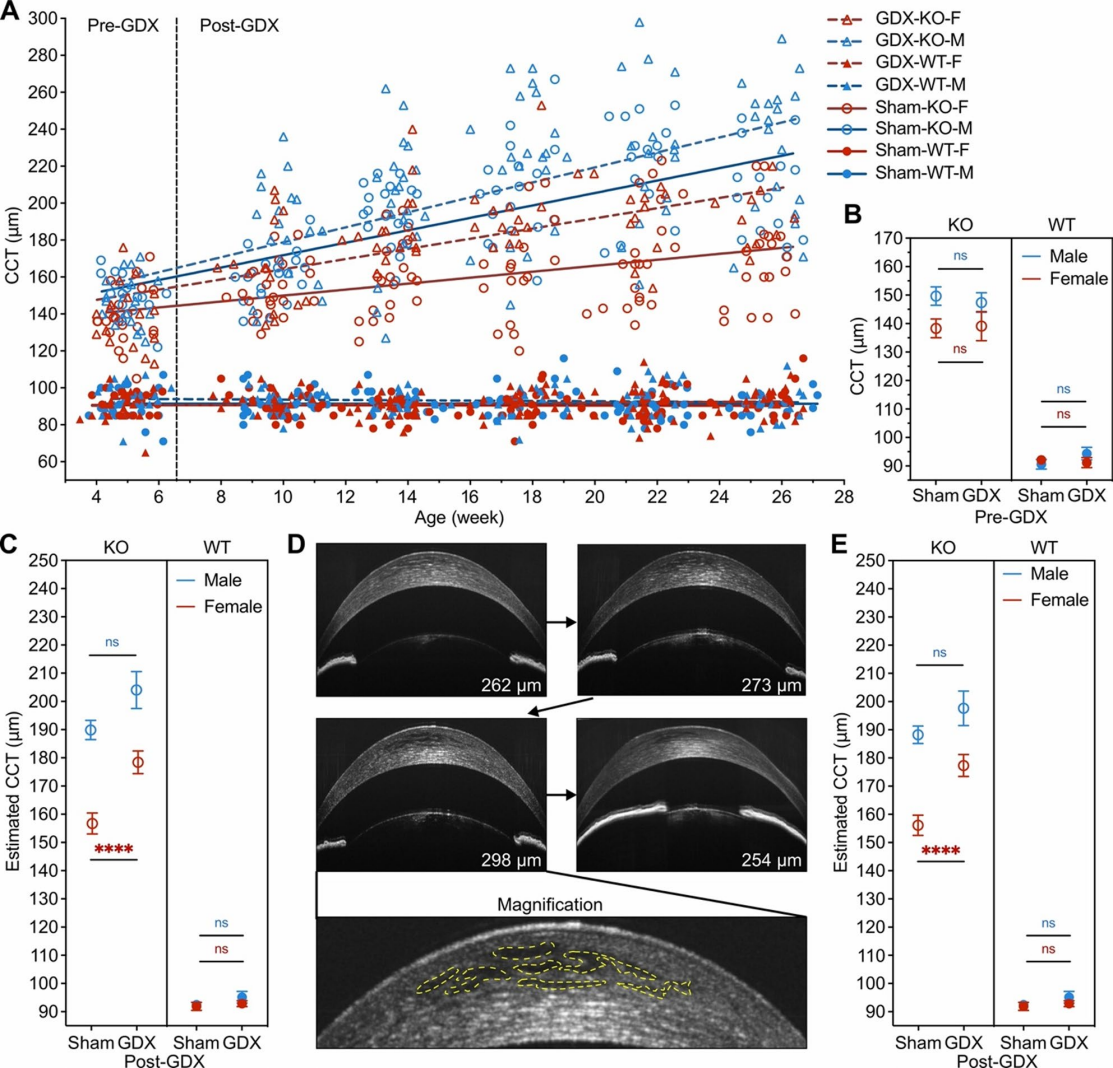
Removal of ovarian hormones in female *Slc4a11*^−/−^ mice worsens corneal edema. (**A**) Scatter plot of CCT measured over time after GDX or sham surgery in KO and WT mice, separated by sex. Fitted linear regression lines are shown for each surgery type, genotype, and sex group. (**B**) Baseline CCT comparison between GDX group versus sham surgery group separated by genotype. (**C**) Bar plot of GLMM model estimated CCT in KO and WT mice at postoperative day 175, separated by genotype and surgery type (GDX vs. sham). (**D**) Representative sequential AS-OCT images of male KO mice after GDX showing the development of transient fluid clefts in anterior 1/3 of corneal stroma. (**E**) Bar plot of GLMM model estimated CCT in KO and WT mice at postoperative day 175, separated by genotype and surgery type (GDX vs. sham), based on the filtered CCT data excluding the CCT values from AS-OCT images clearly showing fluid clefts. ns, *p* > 0.05; ****, *p* < 0.0001

To investigate whether gonadal sex hormones play a role in inducing the apparent sex differences in corneal edema in *Slc4a11*^−/−^ mice, we performed gonadectomy (GDX) at the age of 4–6 weeks in KO mice and WT littermate controls and monitored postoperative CCT changes monthly over a period of 20 weeks (Fig. 3A). GDX was performed in 19 KO mice and 22 WT mice, while sham surgery in which an incision was made without the removal of gonads was performed in 22 KO mice and 20 WT mice. A balanced number of male and female mice were included in each group (Table 1).

**Table 1:** ***Slc4a11***^***+/+***^ **and** ***Slc4a11***^***-/-***^ **mice that underwent gonadectomy (GDX) or sham surgery**

No. of mice	Male	Female	Total
*Slc4a11*^*−/−*^ (KO)			
Total	19	22	41
Sham	9	13	22
GDX	10	9	19
*Slc4a11*^*+/+*^ (WT)			
Total	21	21	42
Sham	10	10	20
GDX	11	11	22

Pre-operative CCT (µm) was significantly higher in male KO mice (148.5 ± 10.3 versus 138.7 ± 13.3, 3-way ANOVA, Genotype, F = 53701, df = 1, *p* < 0.0001; Sex, F = 573.9, df = 1, *p* = 0.14, Genotype*Sex interaction, F = 408.1, df = 1, *p* = 0.037; post-hoc multiple comparison with correction controlling the False Discovery Rate being 0.05, KO male vs. KO female, adjusted *p* = 0.011), while no difference was observed between WT male and female mice (92.5 ± 6.4 versus 91.6 ± 5.4, post-hoc multiple comparison with correction controlling the False Discovery Rate being 0.05, Male KO vs. Female KO, adjusted *p* = 0.52). There was also no difference in the preoperative CCT between the GDX and sham surgery groups in any genotype-sex sub-group (Fig. 3B, 3-way ANOVA, Surgery type (GDX vs. Sham), F = 2.3, df = 1, *p* = 0.87). Monthly postoperative CCT measurements were fitted in a generalized linear mixed model (GLMM) to estimate the effect of GDX on CCT over time among different genotype-sex subgroups (Fig. 3A). The GDX-KO-Female group demonstrated a significantly accelerated increase in CCT over time compared to the Sham-KO-Female group (GLMM, Genotype*Surgery_Type, F = 11.8, df1 = 1, df2 = 458, *p* = 0.001; Genotype*Sex*Surgery_Type, F = 25.5, df1 = 2, df2 = 458, *p* < 0.0001; post-hoc Sidak pairwise comparison of estimated CCT at 25 weeks, GDX-KO-Female vs. Sham-KO-Female, adjusted *p* < 0.0001), with the slope deviating towards the slope of CCT increase in the Sham-KO-Male group, suggesting the loss of ovarian hormones as being responsible for the change. The change in CCT over the 20-week postoperative period in the GDX-KO-Male group was not significantly different than that in the Sham-KO-Male group (post-hoc Sidak pairwise comparison of estimated CCT at 25 weeks, GDX-KO-Male vs. Sham-KO-Male, adjusted *p* = 0.054), indicating that the loss of testicular hormones did not impact CCT. The effect of testicular secretions is likely minimal as the interaction between sex and GDX was also significant (GLMM, Sex*Surgery_Type, F = 28.9, df1 = 2, df2 = 458, *p* < 0.0001), suggesting that the effect of GDX was different in the two sexes. However, the increase in CCT following gonadectomy in the GDX-KO-Female and GDX-KO-Male groups remained significantly different (post-hoc Sidak pairwise comparison of estimated CCT at 25 weeks, GDX-KO-Female vs. GDX-KO-Male, adjusted *p* = 0.001), indicating that the sex difference is not entirely explained by effects of ovarian secretions. Furthermore, GDX or sham surgery did not change the CCT in the WT groups (GLMM, post-hoc Sidak pairwise comparison of estimated CCT at 25 weeks, GDX-WT-Male vs. Sham-WT-Male, adjusted *p* = 0.14; GDX-WT-Female vs. Sham-WT-Female, adjusted *p* = 0.45). The GLMM model-estimated CCT at 25 weeks of age were 91.9 ± 1.4 (Sham-WT-Female), 94.0 ± 1.1 (GDX-WT-Female), 93.6 ± 0.8 (Sham-WT-Male), 96.4 ± 2.1 (GDX-WT-Male), 156.4 ± 3.7 (Sham-KO-Female), 178.0 ± 4.0 (GDX-KO-Female), 189.4 ± 3.4 (Sham-KO-Male), and 203.5 ± 6.5 (GDX-KO-Male) (Fig. 3C).

Anterior segment OCT images used for CCT measurement revealed transient intrastromal fluid clefts in the anterior 1/3 of the corneal stroma in some KO mice (Fig. 3D), raising concern regarding the overestimation of CCT at these time points. Therefore, we excluded CCT data points obtained from images with intrastromal fluid clefts and re-analyzed the CCT data in a GLMM model. The increase in CCT following surgery in the GDX-KO-Male and the Sham-KO-Male groups was not significantly different (GLMM, post-hoc Sidak pairwise comparison of estimated CCT at 25 weeks, adjusted *p* = 0.164), while the increase in CCT following gonadectomy in the GDX-KO-Male and GDX-KO-Female groups was significantly different (GLMM, post-hoc Sidak pairwise comparison of estimated CCT at 25 weeks, adjusted *p* = 0.005). The GLMM model-estimated CCT at 25 weeks of age, excluding these data points, were 156.1 ± 3.6 (Sham-KO-Female), 177.3 ± 3.9 (GDX-KO-Female), 188.2 ± 3.1(Sham-KO-Male), and 197.6 ± 6.1 (GDX-KO-Male) (Fig. 3E).

### Ovarian hormone-mediated enhanced lipolysis in adipose tissue for corneal endothelial β-oxidation may be the cause of the sex difference in CHED and *Slc4a11*^−/−^ mice

**Fig. 4:**
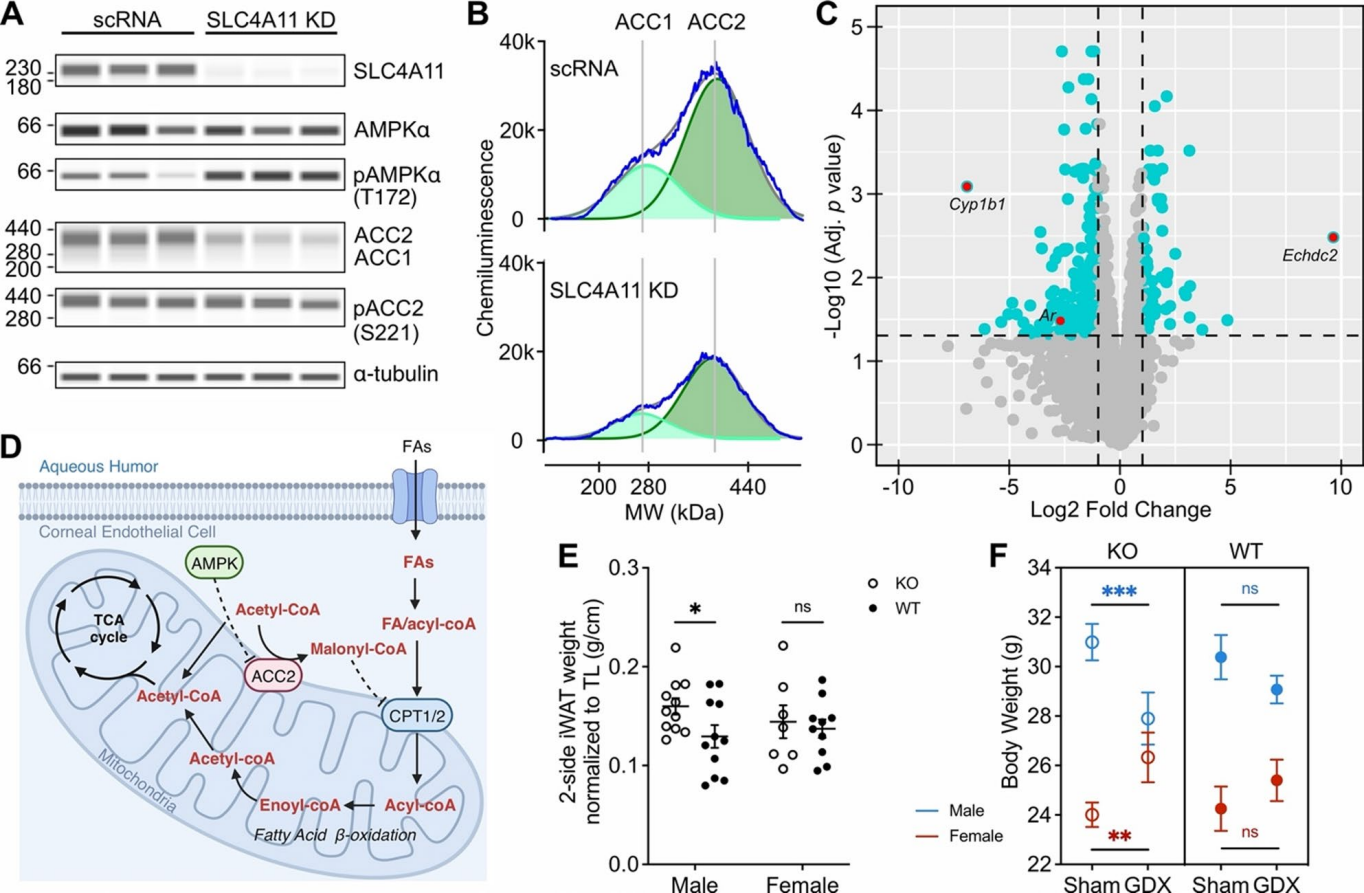
Increased fatty acid β-oxidation in corneal endothelium may be supported by estrogen-mediated increase of peripheral lipolysis. **(A)** Capillary electrophoresis-based Western Blot of AMPKα, pAMPKα (T172), ACC1/2, pACC2 (S221) levels in pHCEC with knockdown of SLC4A11 (SLC4A11 KD) compared to control pHCEC treated with scrambled RNA (scRNA). **(B)** Electropherograms of ACC1/2 bands from (A), showing decreased intensities and peak areas of ACC1 and ACC2 bands in pHCEC with SLC4A11 KD compared to scRNA. **(C)** Volcano plot of identified differentially expressed genes in RNAseq comparing CEC of *Slc4a11*^−/−^ mice to CEC of *Slc4a11*^+/+^ mice, with genes meeting the threshold of |log_2_FC|>1 and adjusted *p* < 0.05 highlighted turquoise and *Cyp1b1*, *Echdc2* and *Ar* highlighted red. **(D)** Schematic of key components regulating fatty acid β-oxidation. **(E)** Dot plot of the weight of bilateral subcutaneous inguinal weight adipose tissue (iWAT) normalized to tibia length (TL) in KO and WT mice separated by sex. **(F)** Bar plot of GLMM model estimated body weight in KO and WT mice at postoperative day 175, separated by genotype and surgery type (GDX vs. sham). ns, *p* > 0.05; *p < 0.05; **p < 0.01; ***p < 0.001

In pHCEC following siRNA knock-down (KD) of SLC4A11, we observed decreased levels of acetyl coenzyme A carboxylase 1 and 2 (ACC1 and ACC2) with increased AMP-activated protein kinase (AMPK)-dependent inhibitory phosphorylation of ACC2 compared to pHCEC treated with scrambled RNA (siRNA) (Fig. 4A and B). Inhibitory phosphorylation of ACC2 leads to inhibition of ACC2 activity and subsequent decreased malonyl-CoA conversion from acetyl CoA, since ACC2 catalyzes such conversion [70]. The decreased malonyl-CoA lessens the inhibition of carnitine palmitoyl CoA transferase 1 and 2 (CPT1/2) and subsequently promotes long-chain fatty acid acyl-CoA import into the mitochondria for β-oxidation [71].

Transcriptomic analysis of CEC from *Slc4a11*^−/−^ mice demonstrated that the most significantly differentially expressed genes compared to CEC from *Slc4a11*^+/+^ mice encode proteins involved in lipid catabolism and estradiol metabolism. Enoyl-CoA hydratase domain-containing protein 2 (*Echdc2*) is the most significantly upregulated gene in CEC from *Slc4a11*^−/−^ mice, with a log_2_FC of 9.6 (Fig. 4C). In mice, the *Echdc2* gene (the ortholog of the human *ECHDC2* gene), encodes one of the catalytic domains of enoyl-CoA hydratase responsible for its enzymatic activity, catalyzing the second step in fatty acid β-oxidation (Fig. 4D) [72]. *Cyp1b1*, the most significantly downregulated gene in CEC from *Slc4a11*^−/−^ mice, with a log_2_FC of −6.9, is the ortholog of the human *CYP1B1* gene. *Cyp1b1* encodes cytochrome P450 (CYP) 1B1 enzyme in mice, which 4-hydroxylates estradiol and decreases its estrogenic activity but also produces free radicals that cause cellular damage [73]. While androgen receptor (*Ar*) expression was decreased 6-fold in CEC from *Slc4a11*^−/−^ mice compared to CEC from *Slc4a11*^+/+^ mice, estrogen receptor (*ER*$\alpha$, *ER*$\beta$) expression was not significantly different (Fig. 4C).

Since there is limited fatty acid storage in ocular tissues, we hypothesized the fatty acid was mobilized from body adipose tissues for corneal endothelial β-oxidation, given estrogen’s well-described role in regulating body fat distribution and suppressing lipogenesis in adipose tissue [74]. Therefore, we performed a comprehensive biometric analysis in male and female *Slc4a11*^−/−^ mice with the comparison to *Slc4a11*^+/+^ mice, which included total body weight, body composition, and weights of liver, bilateral kidneys, spleen, gonadal white adipose tissue (gWAT), subcutaneous inguinal white adipose tissue (iWAT), and brown adipose tissue (BAT). Among all parameters measured, only iWAT showed significantly increased weight in *Slc4a11*^−/−^ male mice compared to *Slc4a11*^+/+^ male mice, while no difference in iWAT weight was observed between female *Slc4a11*^−/−^ and *Slc4a11*^+/+^ mice (Fig. 4E and Supplemental Fig. 2). The male *Slc4a11*^−/−^-specific increase of iWAT suggests that lipolysis of adipose tissue was either reduced in male *Slc4a11*^−/−^ mice or was increased in female *Slc4a11*^−/−^ mice when compared to their sex-matched *Slc4a11*^+/+^ littermates. To test this hypothesis, we analyzed the biweekly postoperative body weight measurements obtained from the previously mentioned GDX experiment in a GLMM to estimate the effect of GDX on body weight over time among different genotype-sex subgroups (Supplemental Fig. 3). Following GDX, the total body weight of *Slc4a11*^−/−^ male mice decreased significantly compared to *Slc4a11*^−/−^ male mice that underwent sham surgery (GLMM, Genotype*Surgery_Type, F = 0.15, df1 = 2, df2 = 921, *p* = 0.864; Genotype*Sex*Surgery_Type, F = 36.3, df1 = 42, df2 = 921, *p* < 0.0001; post-hoc Sidak pairwise comparison of estimated body weight at 25 weeks, GDX-KO-male vs. Sham-KO-male, adjusted *p* = 0.005), while the total body weight of *Slc4a11*^−/−^ female mice increased significantly following GDX compared to *Slc4a11*^−/−^ female mice that underwent sham surgery (Fig. 4F, post-hoc Sidak pairwise comparison of estimated body weight at 25 weeks, GDX-KO-female vs. Sham-KO-female, adjusted *p* = 0.014). In fact, there is no difference in body weights between GDX-KO-male and GDX-KO-female mice (post-hoc Sidak pairwise comparison of estimated body weight at 25 weeks, GDX-KO-male vs. GDX-KO-female, adjusted *p* = 0.210). Such body weight change was not observed in either male or female *Slc4a11*^+/+^ mice after GDX compared to *Slc4a11*^+/+^ mice after sham surgery (Fig. 4F and Supplemental Fig. 2, post-hoc Sidak pairwise comparison of estimated body weight at 25 weeks, GDX-WT-male vs. Sham-WT-male, adjusted *p* = 0.143; GDX-WT-female vs. Sham-WT-female, adjusted *p* = 0.259), consistent with our hypothesis that gonadal hormones play important roles in regulating adipose tissue lipolysis in *Slc4a11*^−/−^ mice.

## Discussion

Our study aims to provide multi-dimensional evidence for sex differences in the corneal phenotype of CHED and in the *Slc4a11*^*−/−*^ mouse model of CHED. This includes sex differences in corneal edema, corneal endothelial mitochondrial superoxide levels, and the corneal endothelial transcriptome. Furthermore, through gonadectomy and body composition analysis in the *Slc4a11*^*−/−*^ mouse model of CHED, we provide evidence supporting the hypothesis that differential levels of ovarian hormone-mediated lipolysis in male and female *Slc4a11*^*−/−*^ mice, due to varying endogenous ovarian hormone levels, may contribute substantially to the sex differences in the corneal phenotype.

We collected the largest cohort of individuals with CHED ever reported in literature, consisting of 177 cases, 104 of whom are previously unreported. In this cohort, male newborns, infants and children demonstrated more severe corneal edema compared with age-matched affected female newborns, infants and children. Prior studies on the clinical features of CHED likely failed to identify this sex difference due to relatively small numbers of affected individuals in each study, lack of consistent measurement and reporting of central corneal thickness and age in affected individuals and historical failure to consider sex as a biologic variable. It is worth noting that no difference in CCT is observed between healthy male and female individuals in the general population [75–78]. The sex difference is recapitulated in *Slc4a11*^−/−^ mice, with the male mice demonstrating significantly greater CCT at 4–6 weeks of age and a greater increase in CCT over the following 20 weeks compared with the female mice. Therefore, the *Slc4a11*^−/−^ mice provide an excellent model to study the pathogenesis and progression of CHED, the factors that account for the sex difference, and a potential sex-dependent response to AAV-SLC4A11 gene therapy, which would be informative for the design of the planned AAV-SLC4A11 gene therapy clinical trial.

Sex differences in *Slc4a11*^−/−^ mice extend beyond the corneal phenotype to mitochondrial superoxide level in CEC and the transcriptome of the CEC. Male *Slc4a11*^−/−^ mice showed a higher level of mitochondria superoxide than in their *Slc4a11*^+/+^ male and *Slc4a11*^−/−^ female counterparts. At the transcriptome level, several genes differentially expressed between male and female *Slc4a11*^−/−^ mice encode proteins that regulate lipid metabolism and that are regulated by sex hormones. The sexually dimorphic differential expression of these genes suggests that the sex difference in CEC function between *Slc4a11*^−/−^ and *Slc4a11*^+/+^ mice may result from differences in lipid metabolism. While comparative analyses of the CEC transcriptome in *Slc4a11*^+/+^ and *Slc4a11*^−/−^ mice have been reported previously, the investigators either performed analysis of whole corneas, with the majority of the extracted RNA coming from the corneal epithelium, or RNA pooled from Descemet membrane and CEC samples from mice without sex information, thus preventing the ability to perform differential expression analyses between male and female *Slc4a11*^−/−^ mice [79, 80]. The decreased expression of androgen receptors with increased expression of estradiol deactivation enzyme CYP1B1 suggest a heightened role of estrogen in CEC of *Slc4a11*^−/−^ mice. Collectively, the results of transcriptomic analysis of CEC from *Slc4a11*^−/−^ mice and Western blot assays in pHCEC following siRNA KD of SLC4A11 are consistent with the hypothesis that, in the setting of loss of SLC4A11 function, there is an upregulation of fatty acid β-oxidation in CEC to compensate for the energy shortage that we have previously demonstrated in CEC of *Slc4a11*^−/−^ mice and pHCEC following KD of SLC4A11 [15].

Gonadectomy performed in *Slc4a11*^−/−^ and *Slc4a11*^+/+^ mice to determine whether gonadal sex hormones are responsible for the difference in CCT between male and female mice revealed that ovarian hormones, estrogen and/or progesterone, is/are primarily responsible for the difference. However, the observation that the magnitude of increase in the CCT in the gonadectomized *Slc4a11*^−/−^ male and female mice remained significantly different is suggestive of additional sex-determined factors other than ovarian hormones in causing the remaining sex difference in CCT in these mice, for example sex chromosome effects. Additional support for this hypothesis is the observation that there was no significant difference in the body weight between gonadectomized *Slc4a11*^−/−^ male and female mice, effectively eliminating a potential role of ovarian hormone-regulated lipid metabolism in causing the residual sex difference in CCT. Interestingly, compelling evidence from Four Core Genotypes (FCG) model links lipid metabolism differences to the XX genotype compared with the XY genotype, potentially due to genes that escape X inactivation and function as epigenetic modifiers influencing metabolic gene networks [81–83]. While the role of estrogen in inducing the sex difference in *Slc4a11*^−/−^ mice is supported by the transcriptome and body composition data, the identification of PGR as one of the top four regulators responsible for the sexually dimorphic differential expression pattern in CEC of *Slc4a11*^−/−^ mice indicates a potential role for progesterone as well. Planned studies to implant either estrogen or progesterone in *Slc4a11*^−/−^ mice to observe the effect on the CCT and CEC transcriptome will provide clarity regarding the role of each of the ovarian hormones in the sex-related differences between male and female *Slc4a11*^−/−^ mice, and presumably between male and female individuals with CHED.

We hypothesize that ovarian hormone-mediated lipolysis in peripheral adipose tissues is increased in female *Slc4a11*^−/−^ mice, mobilizing more free fatty acids to the anterior chamber to be up taken by CEC for β-oxidation, thus providing protection against mitochondria superoxide generation by providing an additional substrate for energy metabolism. Future studies of the uptake of free fatty acids by the CEC in gonadectomized and non-gonadectomized male and female *Slc4a11*^−/−^ mice are planned to investigate this hypothesis. We further hypothesize that following GDX in *Slc4a11*^−/−^ female mice, the loss of ovarian hormone-mediated lipolysis in peripheral adipose tissues is responsible for the observed significant increase in body weight of *Slc4a11*^−/−^ female mice compared to *Slc4a11*^−/−^ female mice that underwent sham surgery. Similarly, in male *Slc4a11*^−/−^ mice, lipolysis activity in peripheral adipose tissues is low, due to low endogenous estrogen levels, leading to the accumulation of subcutaneous fat. However, we propose that the removal of androgen in *Slc4a11*^−/−^ male mice through GDX results in an increase in the ratio of endogenous estrogen to androgen, leading to increased lipolysis and the observed significant decrease in body weight of *Slc4a11*^−/−^ male mice compared to *Slc4a11*^−/−^ male mice that underwent sham surgery.

Lipid metabolism and body weight have also been recently linked to the well-characterized sex differences between men and women with Fuchs endothelial corneal dystrophy (FECD), specifically that female sex is associated with a higher incidence of and more advanced stage of FECD compared to age-matched men [84]. A higher Body mass index (BMI) has been associated with a decreased risk of FECD in women in a recent study, although it has also been associated with a more advanced clinical grade of FECD in women, but not in men, in another study [85, 86]. As no association between BMI and the *TCF4* CTG18.1 expansion was identified, BMI appears to influence the phenotype of FECD through a separate mechanism [85]. A higher incidence of hyperlipidemia has also been reported in individuals with FECD compared to healthy controls, although the authors did not attempt to correlate the severity of hyperlipidemia with that of FECD [87]. Therefore, the data we present regarding CHED, together with published data on FECD, suggest that whole-body lipid metabolism may be an independent modifier of disease severity in corneal endothelial dystrophies. However, the fact that pubertal status, body weight, and plasma lipid levels were not included in the information collected from children with CHED limits our ability to correlate these findings regarding lipid metabolism in *Slc4a11*^−/−^ mice with those in affected individuals. Estrogen and androgen signaling have pleiotropic effects on many pathways involved in lipid metabolism, and these effects may play a crucial role in inducing sex differences in the corneal endothelial dystrophies [88].

## Supplementary Information

Supplementary Material 1.

## Data Availability

Values for all data points in graphs are reported in the Supporting Data Values file. The generated FASTQ files and quantitative results are available from the Gene Expression Omnibus (GEO) database with accession number [GSE288029](https:/www.ncbi.nlm.nih.gov/geo/query/acc.cgi? acc=GSE288029).
