# Supplementary material for: Sex differences in congenital hereditary endothelial dystrophy (CHED) and Slc4a11−/− mouse model of CHED
Source: Biol Sex Differ. 2026 Mar 28;17:99. doi: 10.1186/s13293-026-00879-9 (PMC13151172; doi:10.1186/s13293-026-00879-9)
Supplement: Supplementary file 1 — Supplementary Material 1. [file 13293_2026_879_MOESM1_ESM.docx]

**SUPPLEMENTAL FIGURES**

**
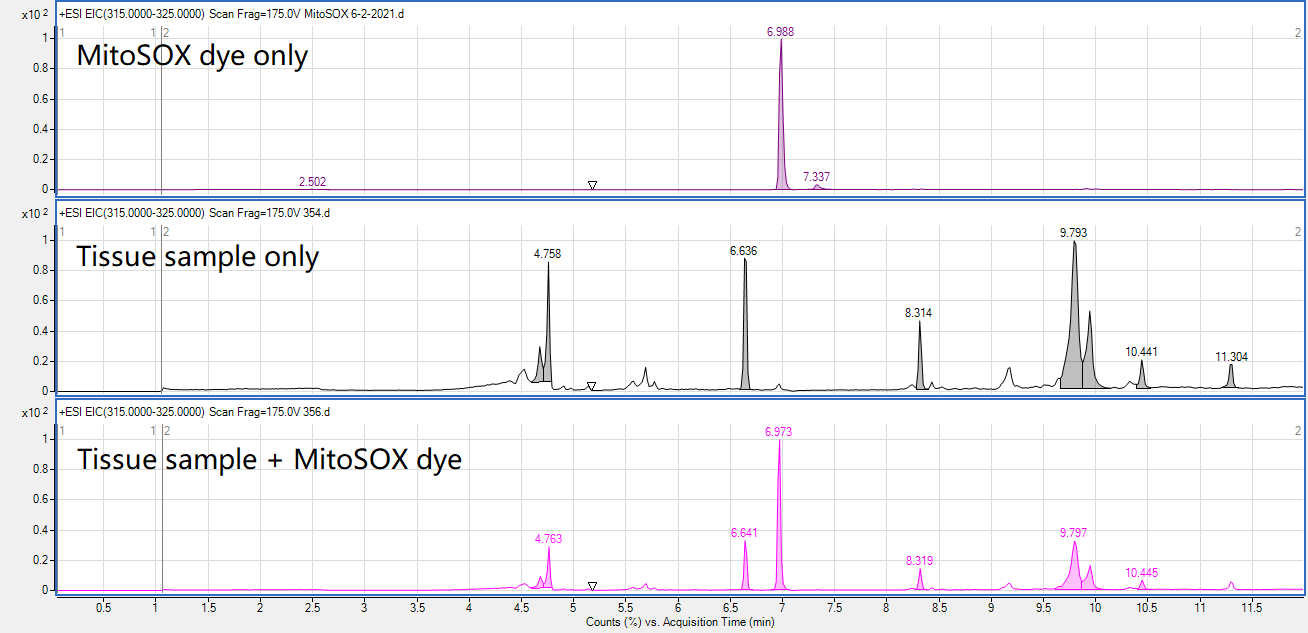
**

**Supplemental Figure 1.** LC-MS chromatograms showing the m/z transitions of samples containing MitoSOX probe only, freshly peeled Descemet membrane tissue only, and Descemet membrane tissue with MitoSOX, showing the retention time of MitoSOX dye at 6.97 min.


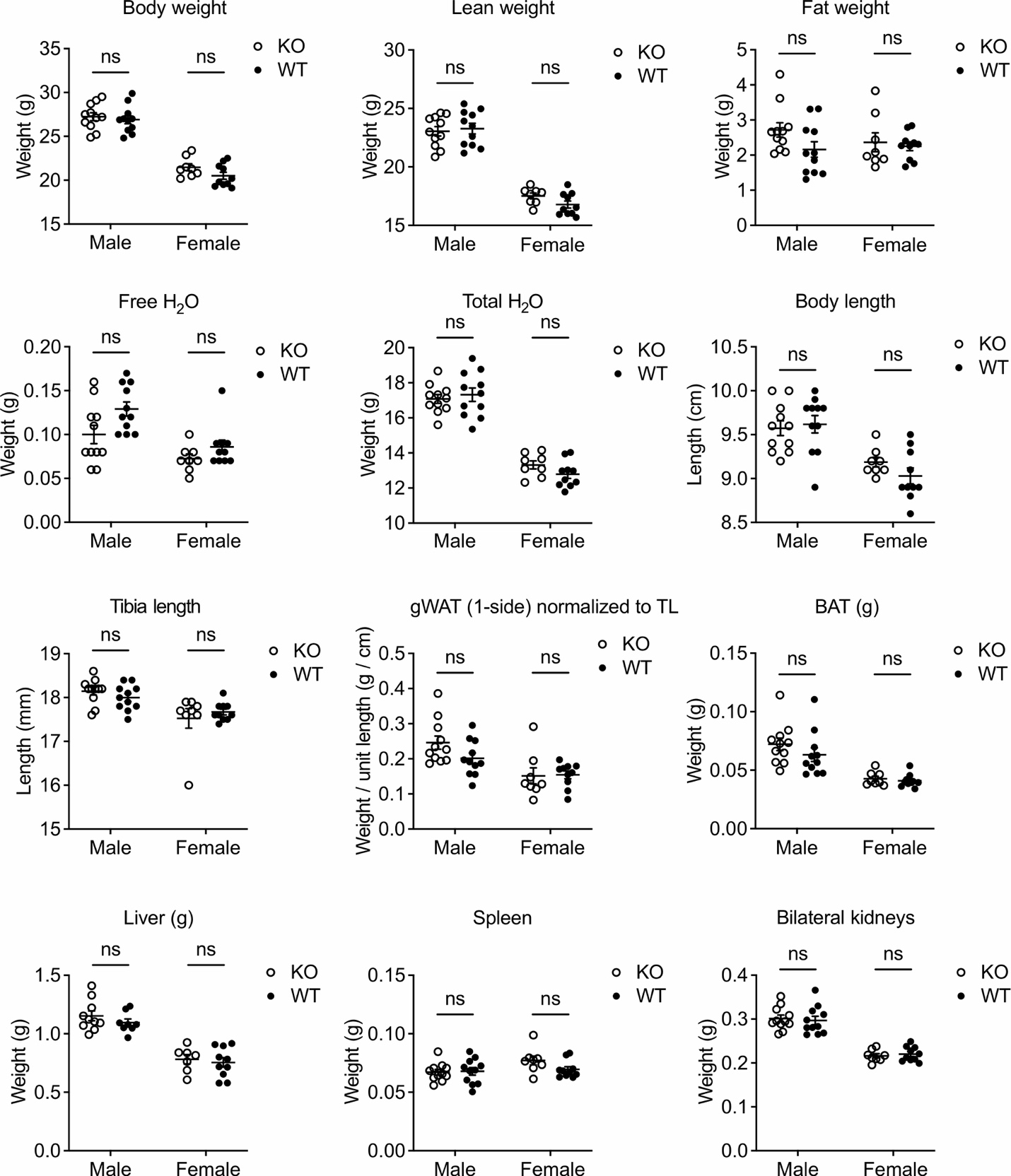


**Supplemental Figure 2.** Dot plots of body weight, NMR-estimated lean muscle weight, NMR-estimated fat weight, NMR-estimated free H_2_O content, NMR-estimated total H_2_O, body length, tibia length, 1-side gonadal WAT normalized to tibia length (TL), brown adipose tissue (BAT), liver weight, spleen weight, and bilateral kidney weight in *Slc4a11*^-/-^ and *Slc4a11*^+/+^ mice separated by sex.

**
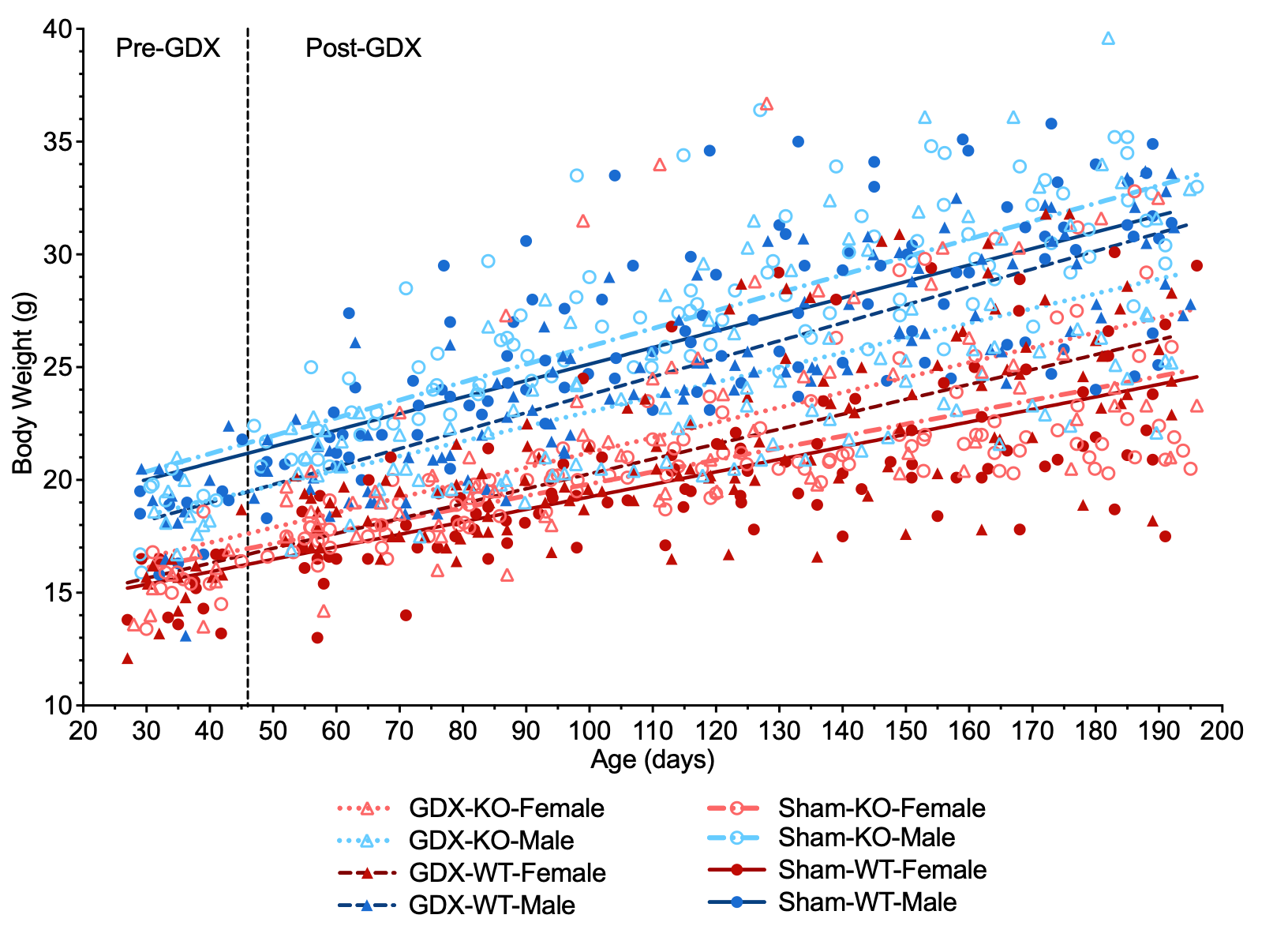
**

**Supplemental Figure 3.** Scatter plot of body weight measured over time after GDX or sham surgery in KO and WT mice, separated by sex. Fitted linear regression lines are shown for each surgery type, genotype, and sex group.

**SUPPLEMENTAL TABLE**

**Supplemental Table 1. Ringer solution composition used for corneal endothelial cell mitochondria superoxide measurement**

|  | **Krebs-Ringer, bicarbonate-buffered**  (Alfa Aesar J67591AP) | **Glutamine-Ringer, bicarbonate-buffered**  (custom) |
| --- | --- | --- |
| NaCl | 120 mM | 120 mM |
| KCl | 5 mM | 5 mM |
| CaCl_2_ | 2 mM | 2 mM |
| MgCl_2_ | 1 mM | 1 mM |
| NaHCO_3_ | 25 mM | 25 mM |
| D-glucose | 5.5 mM |  |
| Glutamine |  | 5.5 mM |
| Osmolarity | 286 mOsm | 285 mOsm |
| pH | 7.3 | 7.3 |
